# Tuning the Morphology of HDPE/PP/PET Ternary Blends by Nanoparticles: A Simple Way to Improve the Performance of Mixed Recycled Plastics

**DOI:** 10.3390/polym14245390

**Published:** 2022-12-09

**Authors:** Angela Marotta, Andrea Causa, Martina Salzano de Luna, Veronica Ambrogi, Giovanni Filippone

**Affiliations:** 1 Dipartimento di Ingegneria Chimica, dei Materiali e della Produzione Industriale (INSTM Consortium–UdR Naples), University of Naples Federico II, P.le Tecchio 80, 80125 Naples, Italy; 2 Pirelli Tyre S. p. A., R&D, Viale Piero e Alberto Pirelli 25, 20126 Milan, Italy

**Keywords:** polymer blends, nanoclay, high density polyethylene, polypropylene, polyethyleneterephtale, morphology, compatibilization, thermal resistance

## Abstract

Due to a very low mixing entropy, most of the polymer pairs are immiscible. As a result, mixing polymers of different natures in a typical mechanical recycling process leads to materials with multiple interfaces and scarce interfacial adhesion and, consequently, with unacceptably low mechanical properties. Adding nanoparticles to multiphase polymeric matrices represents a viable route to mitigate this drawback of recycled plastics. Here, we use low amounts of organo-modified clay (Cloisite^®^ 15A) to improve the performance of a ternary blend made of high-density polyethylene (HDPE), polypropylene (PP), and polyethylene terephtalate (PET). Rather than looking for the inherent reinforcing action of the nanofiller, this goal is pursued by using nanoparticles as a clever means to manipulate the micro-scale arrangement of the polymer phases. Starting from theoretical calculations, we obtained a radical change in the blend microstructure upon the addition of only 2-wt.% of nanoclay, with the obtaining of a finer morphology with an intimate interpenetration of the polymeric phases. Rather than on flexural and impact properties, this microstructure, deliberately promoted by nanoparticles, led to a substantial increase (>50 °C) of a softening temperature conventionally defined from dynamic-mechanical measurements.

## 1. Introduction

The amount of plastic commodities produced since the 1950s, i.e., since the first synthetic polymers began to be produced on an industrial scale, is steadily increasing. Today, plastics are ubiquitous, and their excellent durability is turning out to be a very serious problem as it caused the accumulation of huge amounts of plastic waste in the environment. Even though a reduction in single-use plastic waste would be advantageous, their replacement with other materials (e.g., paper or bioplastics) is not always truly sustainable [1]. In recent years, legislation is regulating the use of single-use plastics [2], encouraging virtuous practices such as reuse and recycling. Aside from being environmentally sustainable, such end-of-life options are also economically advantageous. A recent report by McKinsey states that “plastics reuse and recycling could generate a profit-pool growth of as much as $60 billion for the petrochemicals and plastics sector” [3]. Focusing on recycling, although chemical routes are gaining increasing attention [4], the simplest way to valorize thermoplastic wastes remains the mechanical reprocessing of post-consumer plastics to give these materials a second life [5]. Unfortunately, recycled plastics exhibits unsatisfactory properties that preclude their full exploitation. This is due to several reasons. First, macromolecules suffer from severe degradation processes when heated and melt processed. Using proper chemicals (e.g., chain extenders) and adopting controlled (re)processing conditions (e.g., accurate drying, inert gases, etc.) can mitigate this phenomenon [6]. However, the major weakness of recycled plastics originates from the heterogeneity of the plastic waste stream they are derived from [7,8]. Even if carefully separated, sorted, and cleaned, recyclable post-consumer plastics consist of mixtures of immiscible polymers with uncertain compositions. Therefore, because of the immiscibility of most polymer pairs, recycled products are characterized by multiple interfaces and scarce interphase adhesion.

A possible solution to enhance the stress transfer across the polymer phases and, hence, the mechanical performance of recycled products is using compatibilizing agents [9]. In general, the latter are copolymers, either added to the blend or formed in situ, which act by three mechanisms: (i) decreasing the interfacial tension, (ii) stabilization against coalescence, (iii) enhancement of the adhesion between the phases [10]. Compatibilizers for blends of polyolefins and polar polymers such as polyethylene terephtalate (PET) are particularly desirable, since these classes of plastics are often found as a consequence of an incorrect sorting of urban waste. The majority of the polymeric waste stream is composed by PET and polyolefins (especially HDPE and, in a lesser extent, PP) [11,12] and products where these materials are combined are extremely common. The PET is separately recycled in a well-established recycling path, generally remaining as residual in the waste stream. Numerous examples of compatibilized blends of PET with polypropylene (PP) or polyethylene (PE) with improved mechanical and thermal properties can be found in literature [13,14,15,16,17]. However, compatibilizing copolymers must be finely tailored to the specific polymer pair [18], and even the mixing protocol plays a role [19]. This makes their use hardly applicable in real contexts. In recent years, nanoparticles (NPs) have also proved their effectiveness [20]. The mechanisms behind their compatibilization efficacy are still debated. We do believe that, rather than a truly compatibilization action exerted at the polymer–polymer interface, nanoparticles substantially alter the blend microstructure by acting on the rheology of either the bulk phases [21,22] or the interfaces [23,24,25]. This makes nanoparticles much less system-specific than copolymers, suggesting their use in the field of recycled plastics. To prove this assertion, in this study we added organo-modified montmorillonite (Cloisite^®^ 15A) to a ternary blend of high-density PE (HDPE), PP, and PET from water bottles. Due to a persistently weak interfacial adhesion among the polymer phases, the nanoparticles did not appreciably improve the room temperature flexural and impact resistance. Nonetheless, the clear morphology refinement induced by the tiny amount of nanoclay (2 wt.%) had a drastic effect on the high-temperature mechanical resistance of the blend, which exhibited self-supporting ability up to 250 °C, i.e., well above the melting temperatures of its polyolefin constituents. This remarkable result can be ascribed to the enhanced interpenetration of the polymer phases, which enabled the full exploitation of the high-melting temperature PET phase despite its minority amount in the blend (25%).

## 2. Materials and Methods

### 2.1. Materials

High-density polyethylene (HDPE M80064 from Sabic^®^, Riyadh, Saudi Arabia), polypropylene (PP, Mosten MA 712 by ORLEN UniPetrol, Litvínov, Czech Republic) and polyethyleneterephtalate (PET) from mixed color flaxes from household waste were used for blends production. Cloisite^®^ 15A (C15A, diameter ≤ 2 µm, d_001_ = 31.5 Å, *ρ* = 1.66 g/cm^3^) from Southern Clay Products Inc. (Gonzales, TX, USA), an organomodified clay containing about 43 wt.% of 2M2HT (di-methyl di-hydrogenated tallow), was used as filler.

### 2.2. Blend Preparation

Ternary HDPE/PP/PET blends were prepared by melt-compounding pre-mixed polyolefin pellets and PET flakes at the weight ratio reported in Table 1, using a co-rotating twin screw extruder (Haake PolyLab System, Thermo Electron Corporation, Waltham, MA, USA). This composition can be considered representative of a generic waste stream [26]. The extrusions were performed setting the following temperature profile: 210°–230°–245°–270°–275°–275°–270°–245°–230°. The constituents were blended at a screw speed of ~120 rpm, which led to residence times in the extruder of about 4 min. The nano-filled blend was prepared by adding 2 wt.% of C15A to the initial mixture of pellets and flakes while using the same extrusion protocol. The actual amount of nanoparticles present in the nanocomposite blend was assessed through thermogravimetry (see Supplementary Material). The molten extrudate was cooled in a water bath and pelletized. The pellets, dried for about 10 h at 90 °C under vacuum, were finally compression-molded by using a P300P Collin hydraulic press at 280 °C and 5 bar. The samples used for subsequent tests were cut from the so-obtained plaques (thickness 2 mm).

### 2.3. Experimental

Thermogravimetric analyses (TGA; mod. Q500 by TA Instruments, New Castle, DE, USA) were performed in an inert atmosphere until 700 °C at a heating rate of 10 °C/min. Three samples per composition were tested. Differential scanning calorimetry (DSC; mod. Q20 by TA Instruments) was performed by subjecting the samples to a heating–cooling–heating cycle in the range 30–300 °C in a nitrogen atmosphere. All ramps were performed at 10 °C/min.

Predictions on blends phase morphology and nano-clay localization were made by resorting to the wetting (*ω*) and spreading (*λ*) coefficients:

(1) $$\omega_{ijk}=\frac{\gamma_{jk}-\gamma_{ik}}{\gamma_{ij}}$$

(2) $$\lambda_{ijk}=\gamma_{jk}-\gamma_{ij}-\gamma_{ik}$$

where *γ_ij_* represent the interfacial tension between the components. The latter were computed from the literature data of surface tension for (*γ*) according to Equation (3) [27]:

(3) $$\gamma_{ij}=\gamma_{i}+\gamma_{j}-4\left(\frac{\gamma_{i}{}^{d}\gamma_{j}{}^{d}}{\gamma_{i}{}^{d}+\gamma_{j}{}^{d}}+\frac{\gamma_{i}{}^{p}\gamma_{j}{}^{p}}{\gamma_{i}{}^{p}+\gamma_{j}{}^{p}}\right)$$

The microstructure of the blends was inspected by scanning electron microscopy (SEM, model Leica 420 from Leica, Wetzlar, Germany). The samples were sputter-coated with gold before observations.

The impact strength of the ternary blends and of its nanocomposite was determined using an Instron Ceast 9050 Charpy test rig (Pianezza, Italy), following the UNI EN ISO 179 standard test method. The impact pendulum is 0.374 m long and weighs 7.31 kg. The specimens (cross section 10 × 3 mm) were tested with an impact energy of 50 J, an impact angle of 150°, and an impact speed of 3.7 m/s.

Flexural tests were conducted using a universal testing machine Instron 3360 in a three-point bending configuration. Three samples were tested for each formulation. The tests were analyzed following the ASTM D 790 standard test method.

A Tritec 2000 DMA apparatus (Mettler Toledo, Columbus, OH, USA) was used to perform dynamic mechanical analyses (DMA) in single cantilever bending mode. The samples were deformed with a displacement of 0.02 mm at a frequency of 1 Hz while heated at 3 °C/min from room temperature until the drop of the storage modulus (E′). Pictures of the samples were taken each 10 °C through the window of the DMA apparatus and then analyzed using the open-source ImageJ^®^ software. The sample softening was quantified by defining a dimensionless parameter *a*, defined as the ratio between the maximum deflection at the center of the specimen and the span between the clamps.

## 3. Results

### 3.1. Morphology Prediction by Thermodynamic Calculations

The microstructure developed during the melt blending of mixtures of immiscible polymers is the result of a complex interplay among numerous parameters, such as composition, viscosity ratio, interfacial tension, shear and elongational stresses experienced during mixing [28,29,30,31]. Predicting the final morphology is difficult even in the case of binary blends, and things get more complex in the case of more than two polymers and/or in the presence of nanoparticles. For unfilled ternary blends, reliable predictions about how the polymer phases will combine can be obtained by invoking the spreading theory, introduced by Harkins in 1922 [32] and successfully used in several studies [33,34,35]. The signs of the spreading coefficients (Equation (2)) allow us to predict the space arrangement of the phases [33]. The spreading coefficients for the unfilled ternary blends and the ternary systems constituted by the three pairs of polymers plus the nanoparticles, evaluated through Equation (2), are resumed in Table 2. The interfacial tensions necessary to compute the *λ_ijk_* values were evaluated using the surface energy data reported in literature [20,36] and summarized in Table S2. The evaluation of the *γ_ij_* was carried out considering an equilibrium condition of all systems at a temperature of 275 °C, which corresponds to the maximum value of the temperature profile used for the extrusion of the materials.

Depending on the combination of positive or negative values for the spreading coefficients is possible to obtain separate dispersion, partial engulfing, or complete engulfing microstructures [30]. The fact that all the λ values are negative indicates that each polymer will expose the interface to both the other ones (partial engulfing). Actually, the small absolute values of $\lambda_{PP,PE,PET}$ and $\lambda_{PE,PP,PET}$ suggest that the PET phase prefers to be wetted by the PP.

The spreading theory can be extended to binary blends filled with the third phase of nanoparticles. In this way, for each polymer pair, one can infer which polymer the nanoparticles will tend to accumulate in (if any). The spreading coefficients for the three pairs of polymers are summarized in Table 3. Again, the interfacial tensions were taken from the literature and extrapolated at the processing conditions.

The analysis of the spreading coefficients indicates that, between the two polyolefins, the filler prefers to be encapsulated by the PP phase, while it tends to accumulate at the polymer–polymer interface when either of the polyolefins and the PET touch each other. This picture is supported also by the wetting coefficients, calculated at 275 °C through Equation (1) and summarized in Table 4: the values comprised between −1 and 1 of *ω_PE,PET,C15A_* and *ω_PP,PET,C15A_* indicate interfacial localization between PET and either of the polyolefins, while *ω_PP,PET,C15A_* < −1 indicates embedding in the PP phase when the filler is disputed by the two polyolefins.

To summarize, the analysis of the signs of the spreading indicates that the three polymers should exhibit a partially engulfed morphology, with each phase being in contact with the other two. Looking at the values of *λ_ijk_*, the PET seems to prefer being wetted by the PP. Concerning the nanoparticles, both the spreading and wetting coefficients indicate a preference of C15A to be wetted by the PP phase when the polymer pair is PE/PP, while in all cases in which PET is one of the two polymers, the filler tends to accumulate at the polyolefin/PET interface. 

### 3.2. Morphological Analyses

The picture that emerged from the analysis of the spreading and wetting coefficients suffers from considerable uncertainty due to several factors. First, the literature data used for the surface tensions can be affected by experimental uncertainty. Second, the extrapolation procedure for computing the interfacial tensions at the processing temperature can lead to substantial errors if the temperature coefficients are not very accurate. Third, the high melt mixing temperatures can induce degradation of the polymers and the nanoparticles, with changes in their surface properties. Forth, the predictions hold for equilibrium conditions, which may not be reached in the case of highly viscous polymers and/or with different melting/crystallization temperatures. Therefore, morphological analyses of the blends in the solid state are necessary for a correct interpretation of the results of the characterization.

**Figure 1:**
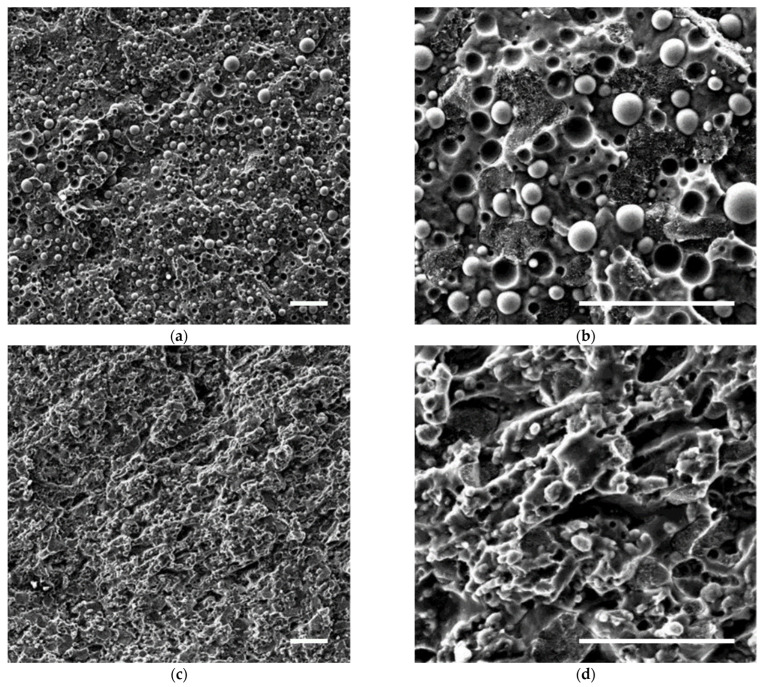
SEM micrographs of (**a**,**b**) unfilled and (**c**,**d**) C15A-filled HDPE/PP/PET blends at different magnification. Scale bars represent 50 µm.

The SEM micrographs of the filled and unfilled blends are shown in Figure 1. In the unfilled system, the minority PET phase forms spherical droplets (volume–average radius 4.4 ± 1.3 µm) suspended in a polyolefin matrix. A closer look reveals that HDPE and PP form a co-continuous microstructure. The two phases are recognizable by the different texture of their fractured surfaces: the more ductile HDPE exhibits corrugated texture due to localized yielding, while the more fragile PP has a smoother appearance. Discriminating between PP and HDPE allows us to notice that the PET droplets are preferentially embedded in the PP phase. Aside from reflecting a preference of PET towards the PP that emerged from the analysis of data in Table 2, the presence locally of completely engulfed regions (PET drops in PP domains) could depend on changes in the surface energy of PET during (re)processing due to the high reactivity of the ester linkages [37]. Overall, the microstructure observed for the unfilled blend appears in good agreement with the predictions based on the spreading coefficients.

The addition of the nanoparticles has a drastic effect on the blend morphology. The most evident changes are (i) the refinement of the microstructure and (ii) the apparent disappearance of the PET phase. Regarding the first point, the characteristic size of the co-continuous domains, defined as the inverse of interfacial length per unit area (see Appendix A) [38], decreases by about 15% in the presence of the filler. It should be mentioned that the identification of the co-continuous domains becomes difficult in the filled blend. This suggests better compatibility among the polymeric phases, which better interpenetrate becoming hardly discernible. Even more evident is the effect of the nanoparticles on the PET phase. The spherical droplets are no longer visible, being apparently replaced by irregularly shaped, micron-sized domains.

Overall, the combination of (i) refinement of the co-continuous pattern, (ii) loss of sphericity of the PET phase, and (iii) uneven distribution of the PET along the PE/PP contours and in the bulk of the PP phase induced by the nanoparticles results in a better interpenetration of the polymer phases. However, the rough texture of the fracture surface of Figure 1d suggests that the filler did not improve the polymer–polymer interfacial adhesion.

### 3.3. Mechanical Behavior at High Temperature: Flexural and Impact Strength

**Figure 2:**
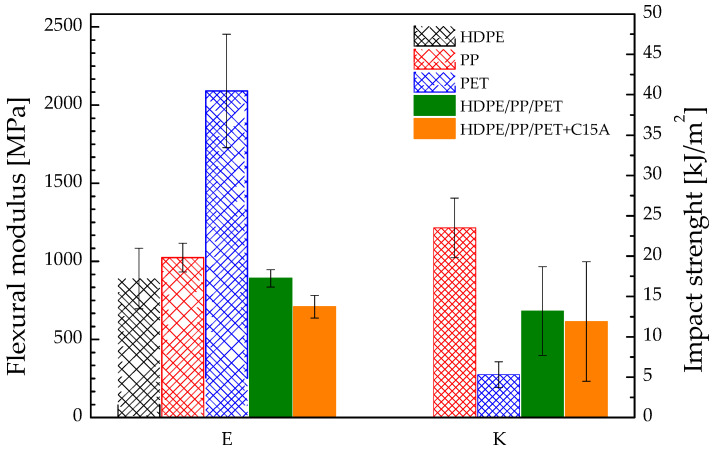
Flexural modulus and impact strength for pure polymers and blends. The impact strength of HDPE is not reported as this sample bent without breaking.

The mechanical properties of polymer blends are strictly correlated to their morphology at the micron scale. In binary blends with co-continuous morphology, nanoparticle-induced morphology refinements lead to a mechanical interlocking of the polymer phases. If one of the polymers exhibits high mechanical strength, phase interpenetration results in enhanced mechanical performance [39]. Here, the system is more complex, and the mechanically strongest phase is the PET, which is a minority component of the blends. Therefore, it is not surprising that neither the flexural modulus (E) nor the impact strength (K) of our materials increases with the addition of nanoparticles (Figure 2). 

In particular, the modulus of the blends is governed by those (similar between them) of the polyolefins, which represent the continuous phase of the materials and, hence, bear the stress. The morphology refinement induced by the nanoparticles does not have a beneficial effect, and the modulus is even lower than that of the unfilled blend. To better understand such unexpected behavior, DSC analyses were performed to verify the effect of the nanoparticles on the degree of crystallinity (*χ*) of the polymer phases. The results are summarized in Table 5.

Blending the polymers without nanoparticles causes a decrease in *χ* for the PP and PET phases. Changes in crystallinity in immiscible blends are common and can be due to several reasons, among which are confinement effects, fractionized crystallization, nucleating action of the interfaces [40]. In the presence of nanoparticles, the crystallinity of the HDPE decreases as well. Being the HDPE a continuous phase of the blends, its weakening because of the impact of the nanoparticles on *χ* is consistent with the slight decrease in the modulus noticed in the presence of the nanoparticles.

Looking at the results of the impact tests, the behavior of the pure polymers ranges between highly ductile (HDPE, which bends without breaking, and PP) and quite brittle (PET). The unfilled blend exhibits intermediate behavior between those of the PP and PET. The impact response reflects the microstructure: it is governed by the continuous polyolefin phases, while the PET phase exerts an embrittlement effect (decrease in K) because of the stress concentration around the (weakly adhering) drops. The decrease in the crystallinity degrees of the PP phase could contribute to some extent to enhancing the impact strength. Surprisingly, the substantial change in the blend morphology upon addition of the nanoparticles does not alter the impact behavior, and the nano-filled blend exhibits the same impact strength as its unfilled counterpart. The decrease in the HDPE crystallinity should contribute to the ductility of the nanocomposite blend, balancing the embrittlement effect generally associated with the presence of nanoparticles [41]. The role of the nanoparticle-induced morphology changes is, however, difficult to quantify.

### 3.4. Mechanical Behavior at High Temperature: DMA Analyses

Although the absence of embrittlement in the presence of nanoparticles is an encouraging result, the mechanical performance at room temperature of the nano-filled blend is quite disappointing. Things change when the comparison is made at high temperatures. 

**Figure 3:**
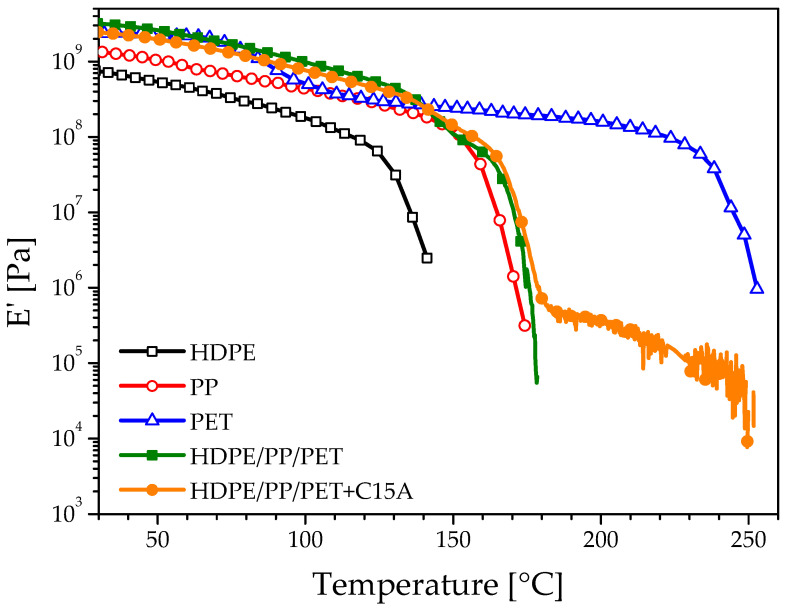
Storage modulus for the neat polymers and the blends as evaluated by DMA.

**Figure 4:**
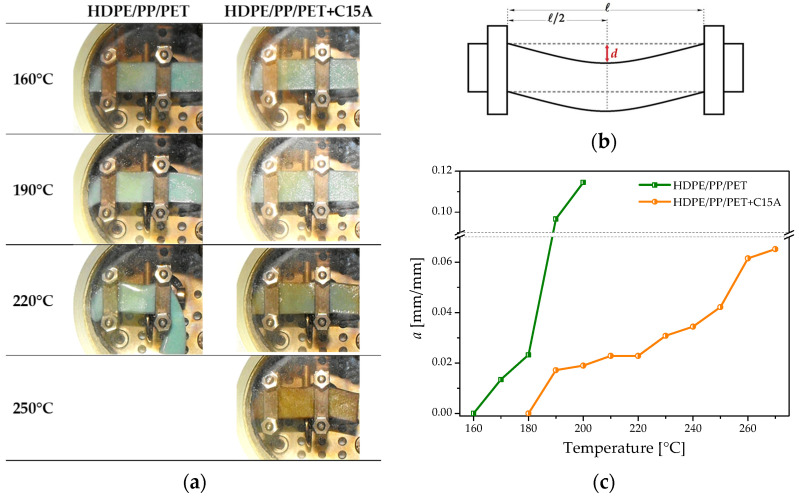
(**a**) Pictures of the specimens taken at different temperatures during the DMA tests. (**b**) Definition of the dimensionless maximum deflection *a = d/l*. (**c**) Temperature dependence of *a* for the unfilled and nano-filled blend.

The temperature dependence of the storage modulus (E′) of neat polymers and blends is reported in Figure 3 as recorded by DMA analysis. The values at 30 °C reflect what is already seen by looking at the flexural modulus. Increasing the temperature each polymer experiences its characteristic decrease in the modulus, with a fall when the melting temperature is reached (Figure 3). The behavior of the blends (Figure 3) provides relevant information about their morphologies. The modulus of the unfilled blend lies in between those of the two polyolefins, confirming the co-continuous morphology of its polyolefin phase [42]. The fall of E′ starts around 140 °C, i.e., when the HDPE melts down, and it completes at the melting of the PP at ~170 °C. The behavior of the nano-filled blend retraces that of the unfilled one, but a noisy signal is still recorded well above the melting of the PP phase. In particular, a modulus of the order of ~10^5^ Pa persists up to about 250 °C, which is close to the melting temperature of the PET phase. This suggests that the PET phase, hardly recognizable in the SEM micrograph (see Figure 1c,d), could have changed from a dispersed morphology to a continuous one, thus contributing to bear a fraction of the load. Representing only 25% of the polymeric fraction, its contribution is small, as indicated by the low modulus (~10^5^ Pa) recorded during the DMA test. Nonetheless, the macroscopic effect of such a morphology change is nothing but negligible. This can be seen in Figure 4a, where pictures of the specimens taken during the DMA tests are collected at different temperatures.

The unfilled blend begins to soften as soon as the PP phase, i.e., the continuous phase with the highest *T_m_*, starts melting down. This sudden softening phenomenon can be appreciated by looking at the evolution of the parameter *a*, defined as the maximum deflection at the center of the specimen divided by the span between the clamps (see Figure 4b) as a function of temperature (Figure 4c). Differently, the nano-filled specimen preserves its self-standing ability up to 250 °C, i.e., is well above the melting of the polyolefin phases, owing to the contribution of the minority yet continuous PET phase. This notable result is ascribable to the synergic combination of the different effects of the nanoparticles on the blend morphology. In particular, the refinement of the polymer phases, the loose of sphericity of the PET phase, and its confinement in specific regions of the material (PP phase and PP/PE interface), concur in making the minority PET phase (25%) effective in providing the blend with superior heat deflection resistance.

## 4. Conclusions

The effect of Cloisite^®^ 15A on the morphology, thermal properties, and mechanical behavior of a ternary HDPE/PP/PET blend with a majority of polyolefins (75%) was investigated. The morphology of the unfilled blend was predicted by calculating the spreading and wetting coefficients. SEM analyses confirmed thermodynamic calculations, showing a propensity for the minority PET phase to enrich the continuous PP phase or the PP/PE interface. The addition of the nanoparticles had a drastic effect on the blend morphology, causing a refinement of the co-continuous microstructure and promoting a fine interpenetration of the three polymer phases, which became hardly distinguishable. This morphological change had a negligible effect on the mechanical behavior at room temperature: neither the flexural modulus nor the impact strength benefited from the reinforcing effect of the nanoparticles and the morphological refinement. This has been ascribed to the inherently scarce mechanical properties of immiscible polymer blends, typically characterized by weak interfacial adhesion among the phases that prevents an effective stress transfer. The morphological refinement has instead a significant influence on the material structural integrity at high temperatures resulting in the preservation of the mechanical resistance up to 250 °C (differently from the unfilled blend that melts at 180 °C).

## Figures and Tables

**Table 1:** Samples composition.

Sample	HDPE [wt.%]	PP [wt.%]	PET [wt.%]	C15A [phr]
HDPE/PP/PET	37.5	37.5	25	0
HDPE/PP/PET	37.5	37.5	25	2

**Table 2:** Spreading coefficients (in mN/m) of the polymer triplets.

$\lambda_{PE,PP,PET}$	−1.09
$\lambda_{PE,PET,PP}$	−10.20
$\lambda_{PP,PE,PET}$	−0.68

**Table 3:** Spreading coefficients (in mN/m) for ternary systems constituted by the three possible polymer pairs plus the nanoparticles.

$\lambda_{PP,PE,C15A}$	−2.85
$\lambda_{PE,PP,C15A}$	1.08
$\lambda_{PE,C15A,PP}$	−10.40
$\lambda_{C15A,PE,PET}$	−10.61
$\lambda_{PE,C15A,PET}$	−2.63
$\lambda_{PE,PET,C15A}$	−0.27
$\lambda_{C15A,\;PP,PET}$	−8.85
$\lambda_{PP,C15A,PET}$	−0.47
$\lambda_{PP,PET,C15A}$	−2.43

**Table 4:** Wetting coefficient for C15A into the polymer pairs.

$\omega_{PE,PP,C15A}$	−2.22
$\omega_{PE,PET,C15A}$	−0.60
$\omega_{PP,PET,C15A}$	−0.90

**Table 5:** Melting and crystallization temperature and degree of crystallinity for the pure polymers and the polymer phases of the blends.

Sample	Phase in the Blend	*T_m_* [°C]	*T_c_* [°C]	*χ* (%)
HDPE		134.4 ± 1.2	117.1 ± 0.7	65.8 ± 1.8
PP		164.4 ± 0. 9	117.9 ± 1.3	40.3 ± 2.4
PET		250.9 ± 0.4	202.5 ± 5.0	28.9 ± 3.1
HDPE/PP/PET	*HDPE*	133.4 ± 0.7	117.7 ± 1.2	66.1 ± 2.0
	*PP*	162.9 ± 0.9		30.7 ± 1.1
	*PET*	244.9 ± 1.5	202.4 ± 2.7	18.2 ± 1.8
HDPE/PP/PET+C15A	*HDPE*	132.6 ± 0.5	114.6 ± 1.0	58.3 ± 3.9
	*PP*	165.0 ± 1.0		29.0 ± 2.7
	*PET*	252.1 ± 0.2	196.6 ± 1.2	21.2 ± 3.4

## Data Availability

The data presented in this study are available on request from the corresponding author.

## Supplementary Materials

The following supporting information can be downloaded at: https://www.mdpi.com/article/10.3390/polym14245390/s1, Table S1: Degradation temperatures at 10% (T_d10_), degradation temperatures at the maximum degradation rate (T_d_) and residue at 600 °C (R_600_), evaluated by TGA analysis; Table S2: Surface energies (γ), dispersive (γ^d^) and polar (γ^p^) components of polymers and nanoclay used in this work.

## Appendix A

Characteristic dimensions of the polymeric phases were determined by the analysis of SEM micrographs, with the help of the ImageJ^®^ software.

For the estimation of the size of the dispersed phase droplets the following parameters were evaluated:

- The number–average drop radius

(A1) $$R_{n}=\sum_{i=1}^{n}\frac{n_{i}}{n_{tot}}R_{i},$$

with *n_i_/n_tot_* number of drops respect to the total, having radius *R_i_*;

- The volume–average drop radius

(A2) $$R_{V}=\sum_{i=1}^{n}\frac{V_{i}}{V_{tot}}R_{i},$$

with *V_i_/V_tot_* volumetric fraction of drops having radius *R*_i_;

- The circularity of a drop of radius *R_i_*, area *A_i_* and perimeter *P_i_*

(A3) $$C_{i}=\frac{2}{R_{i}}\cdot \frac{A_{i}}{P_{i}},$$

which is 1 for a perfectly spherical drop (circular in two dimensions) and decreases as we move away from this geometry;

- The *R_v_/R_n_* ratio, called polydispersity, which is an index of the amplitude of the drop size distribution.

An estimate of the characteristic size of the continuous phase domain was, instead, given by the ξ factor, obtained by the analysis of the SEM micrographs. ξ was defined the inverse of interfacial length per unit area [38].
