# Supplementary material for: Tuning the Morphology of HDPE/PP/PET Ternary Blends by Nanoparticles: A Simple Way to Improve the Performance of Mixed Recycled Plastics
Source: Polymers (Basel). 2022 Dec 9;14(24):5390. doi: 10.3390/polym14245390 (PMC9782910; doi:10.3390/polym14245390)
Supplement: Supplementary file 1 [file polymers-14-05390-s001.zip › polymers-2072770-supplementary.pdf]

Supplementary material

Tuning the Morphology of HDPE/PP/PET Ternary Blends by Nanoparticles: A Simple Way to Improve the Performance of Mixed Recycled Plastics

Angela Marotta [1], Andrea Causa [2], Martina Salzano de Luna [1], Veronica Ambrogi [1] and Giovanni Filippone [1,*]

[1] Dipartimento di Ingegneria Chimica, dei Materiali e della Produzione Industriale (INSTM Consortium–UdR Naples), University of Naples Federico II, P.le Tecchio 80, 80125 Naples, Italy
[2] Pirelli Tyre S. p. A., R&D, Viale Piero e Alberto Pirelli 25, 20126 Milan, Italy
* Correspondence: gfilippo@unina.it

To verify the presence of C15A into the nanofilled blend and its precise amount thermogravimetric analysis have been conducted. TGA analysis on the nanofiller gives us information about the thermal resistance of the filler and the exact amount of organomodifier. As can be deduced from data reported in Table S1, about the 45 wt.% of the nanoclay is chargeable to the organomodification, and approximatively the 10 wt.% of C15A is lost at the process temperature. In the light of this, and considering the difference among the residues of filled and unfilled blend, TGA analysis confirms that almost 2 wt.% of C15A is present in the HDPE/PP/PET+C15A blend.

Table S1. Degradation temperatures at 10% (T_{d10}), degradation temperatures at the maximum degradation rate (T_d) and residue at 600 °C (R_{600}), evaluated by TGA analysis.

Sample	T_{d10} [°C]	T_d [°C]	R_{600} [%]
C15A	275.0 ± 5.3	312.8 ± 0.3	56.5 ± 0.6
HDPE/PP/PET	413.6 ± 2.0	472.3 ± 1.0	3.3 ± 0.3
HDPE/PP/PET+C15A	410.5 ± 6.2	476.3 ± 2.8	4.9 ± 0.6

Table S2. Surface energies (γ), dispersive (γ^d) and polar (γ^p) components of polymers and nanoclay used in this work.

Sample	γ [mN/m]	γ_d [mN/m]	γ_p [mN/m]	dγ/dT [mN/m°K]	T_{ref} [°C]
HDPE	25.9	25.9	0	-0.057	190
PP	15.7	15.7	0	-0.065	270
PET	24.35*	18.95*	5.4*	-0.058	270
C15A	18.5	13.7	4.8	-0.129	260

[1] Values here reported are the mean of values reported in literature. [18]

Values here reported for surface energy (and its dispersive and polar components) for PET are the mean of values reported in literature, since, it is necessary to remember, a recycled material was used; therefore, composed of PET of a different nature.

References

20. Taguet, A., Cassagnau, P., Lopez-Cuesta, J.M. Structuration, selective dispersion and compatibilizing effect of (nano) fillers in polymer blends. *Prog. Polym. Sci.* **2014**, *39(8)*, 1526-1563
